# Overexpression of CENPF is associated with progression and poor prognosis of lung adenocarcinoma

**DOI:** 10.7150/ijms.49041

**Published:** 2021-01-01

**Authors:** Mei-Xiang Li, Meng-Yu Zhang, Huan-Huan Dong, Ai-Jun Li, Hai-Feng Teng, Ai-Ling Liu, Ning Xu, Yi-Qing Qu

**Affiliations:** 1 Department of Pulmonary and Critical Care Medicine, Qilu Hospital, Cheeloo College of Medicine, Shandong University, Jinan 250012, China.; 2 Department of Respiratory Medicine, Weihai Municipal Hospital, Weihai 264200, China.; 3 Department of Pathology, Weihai Municipal Hospital, Weihai 264200, China.; 4 Department of Pulmonary and Critical Care Medicine, Qilu Hospital of Shandong University, Jinan 250012, China.

**Keywords:** CENPF, LUAD, prognostic biomarker, cell cycle

## Abstract

**Background and aim:** The molecular signatures of lung adenocarcinoma (LUAD) are not well understood. Centromere protein F (CENPF) has been shown to promote oncogenesis in many cancers; however, its role in LUAD has not been illustrated. We explored the role of CENPF in LUAD.

**Methods:** CENPF expression level was investigated in public online database firstly, the prognosis of CENPF in LUAD were also assessed by Kaplan-Meier analysis. Then quantitative reverse transcription-polymerase chain reaction (qRT-PCR) was performed using 13 matched pairs of clinical LUAD tissue samples. Subsequently, the impact of CENPF expression on cell proliferation, cell cycle, apoptosis, colony formation was investigated by 3-(4,5-dimethylthiazol-2-yl)-2,5-diphenyl tetrazolium bromide (MTT), flow cytometric analysis and colony formation assay, respectively. Finally, experimental xenograft lung cancer model of nude mice armpit of right forelimb to determine the effect of CENPF on LUAD tumorigenesis.

**Results:** CENPF mRNA expression was significantly elevated in LUAD tissues compared with adjacent non-tumor lung tissues in Gene Expression Profiling Interactive Analysis (GEPIA) (*P* < 0.001). Up-regulated CENPF was remarkably positively associated with pathological stage, relapse free survival (RFS) as well as overall survival (OS) of LUAD patients. Besides, CENPF knockdown greatly suppressed A549 cell proliferation, induced S phase arrest, promoted apoptosis and decreased colony numbers of LUAD cells. Furthermore, knockdown of CENPF significantly inhibited the tumor growth of the LUAD cells in an experimental xenograft lung cancer model of nude mice armpit of right forelimb.

**Conclusion:** Taken together, these results demonstrated that CENPF may serve as a potential biomarker of prognostic relevance and a potential therapeutic target for LUAD.

## Introduction

Lung cancer is still the most common reason of cancer-related mortality worldwide, and nearly 80% are non-small cell lung cancer (NSCLC) 1,2. Lung adenocarcinoma (LUAD) is the main subtype of NSCLC. However, the molecular basis of the pathogenesis of LUAD is still not well elucidated. Despite there are many new modalities of treatment appeared, the 5-year survival rate of advanced LUAD is less than 20%, while the 5-year survival rate of stage IA LUAD can reach 60% 3. In recent years, some biomarkers with certain diagnostic and prognostic value have been confirmed in basic research and expected to be included in routine clinical diagnosis and treatment 4,5. Hence, improve the prognosis through early diagnosis with specific biomarkers and identification of novel treatment targets are important for the control of LUAD.

Centromere protein F (CENPF) gene is located on chromosome 1q41 and encodes for CENPF, which acts as part of the centromere kinetochore complex and a component of the nuclear matrix during G2 of interphase 6-8. CENPF expression is in a cell cycle-dependent manner, gradually accumulated during the process of cell cycle, then reached peak levels in G2/M phase, finally degraded upon completion of mitosis 9. Expression changes of CENPF have been shown to correlate with the prognosis of various cancers. Functionally speaking, cell proliferation and apoptosis are known to be affected by CENPF 10. Correlation of CENPF overexpression with metastasis, TNM stage and poor prognosis has been demonstrated in the context of bladder and breast cancers 11, 12. However, the impact of CENPF in LUAD has not been well studied. In the current study, we explored the relationship among CENPF expression level, different pathological stages and prognosis in patients with LUAD. Furthermore, the effect of CENPF on proliferation, apoptosis and cell cycle of LUAD cells were also assessed *in vitro* and *in vivo*.

## Materials and Methods

### Validation the expression level and prognostic value of CENPF

Publicly available database for NSCLC, lung squamous cell carcinoma (LUSC) and LUAD were searched in the Gene Expression Profiling Interactive Analysis (GEPIA) (http://gepia.cancer-pku.cn/) to analyze CENPF mRNA expression between tumor and adjacent non-tumor lung tissues. Gene expression profiles (GSE3141, GSE8894, GSE4573, GSE17710 and GSE31210) for NSCLC, LUSC and LUAD were also searched in the Gene Expression Omnibus (GEO) database for prognostic analysis.

### Patients and samples

From December 2019 to January 2020, a total of 13 pairs of fresh LUAD and adjacent non-tumor lung tissues (5cm distant from lung cancer) were obtained during surgical resection. All these 26 lung tissue samples were enrolled after signed informed consent form in Weihai Municipal Hospital of Shandong Province. All patients did not undergo any chemotherapy or radiation therapy prior to operation, and had no other malignancies accompanied. Of these samples, 13 pairs of LUAD tissues and adjacent non‑tumorous tissues were frozen immediately, stored at ‑80 °C and used for quantitative reverse transcription-polymerase chain reaction (qRT-PCR) analysis. This study was approved by the ethics committee of Weihai Municipal Hospital (2020024).

### RNA isolation and reverse transcription

Total RNA was extracted from A549, H1299, H446 and H69 cell lines and the patients' lung tissues using TRIzol Reagent (Invitrogen) according to the manufacturer's instructions. Then, the purity and concentration of all extracted total RNA were tested and total RNA was subjected to cDNA synthesis using the PrimeScript RT Reagent Kit (Takara). Finally, 1000 ng total RNA was reverse transcribed into cDNA with Oligo (dT) primers in a final volume of 20 μL.

### qRT-PCR

The qRT-PCR was performed using the TB Green Premix Ex Taq II (TaKaRa) on the Applied Biosystems StepOnePlus Real-Time PCR System (Thermo Fisher Scientific). Through this experiment, Glyceraldehyde 3-phosphate dehydrogenase (GAPDH) was used as a reference gene and the divergent primers for CENPF and GAPDH were obtained from BioSune Corporation (Shanghai, China). Sequences of primers used for qRT-PCR are as follows: GAPDH, forward primer 5′‐TGACTTCAACAGCGA CACCCA-3′, reverse primer 5′-CACCCTGTTGCTGTAGCCAAA-3′. CENPF, forward primer 5′-AGCACGACTCCAGCTACAAGGT-3′, reverse primer 5′-CATCA TGCTTTGGTGTTCTTTCTG-3′. The PCR conditions were set as the followings: 95 °C, 30 s; followed by 40 cycles at 95 °C, 5 s; finally, 60 °C, 30 s for every specific primer. In the end, melting curves were generated to ensure the specificity of PCR. Finally, the relative expression levels of CENPF were calculated using the 2^-ΔΔCT^ method.

### Cell culture

All cell lines (A549, H1299, H446 and H69) were purchased from Cell bank, Shanghai institute of life sciences, Chinese academy of sciences, and confirmed by short tandem repeat (STR) profiling. A549 and H1299 are LUAD cell lines, H446 and H69 are SCLC cell lines. All of these cell lines were cultured in RPMI-1640 medium (Gibco), supplemented with 10 % fetal bovine serum (Biological Industries) and 1% penicillin/streptomycin (Gibco). All cell lines were grown at 37 °C with 5% CO_2_.

### Cell proliferation assay

To measure whether CENPF was involved in cell proliferation, we performed the MTT assay. A549 cells were seeded into 96-well plates at a density of 5×10^3^/well and cultured for 24 h after transfection. Cell proliferation was assessed using the MTT Cell Growth Assay Kit (Sigma-Aldrich) according to the manufacturer's instructions. The optical density was measured by a microplate reader set at 490 nm. In order to control the experimental data error, the experiments were performed at least three times.

### Plasmid construction and lentivirus transfection experiment

The siRNA (si-CENPF, 5′-GCGCAGAAUCAAGAGCUAA-3′) was obtained from RiboBio (Guangzhou, China). The A549 cells were transfected with siRNA using the Lipofectamine 2000 Reagent (Invitrogen) according to the manufacturer's instructions. After transfection, the cells were processed to assess the knockdown activity by qRT-PCR. Lentiviral vectors encoding the human CENPF gene were purchased from GeneChem (Shanghai, China) for further functional studies. CENPF silence via lentiviral transduction with specific shCENPF vectors (GeneChem), the negative control duplex with a scramble sequence. The following operation were conducted according to the manufacturer's instructions. Sequences of the interference targets are the followings: shCENPF: 5′-AGCACGACTCCAGCTACAAGGT-3′. shCtrl: 5′-TGACTTCAACAGCGACACCCA-3′.

### Flow cytometry assay

Cell cycle and apoptosis was determined by flow cytometry analysis. A549 cells were harvested 96 h after transfection and washed with cold D-Hanks (pH=7.2-7.4). Then cells were fixed with 70% ethanol and placed at 4 °C overnight. The cells were resuspended in 5 μL staining solution (PI/RNase) (Sungene) for 30 min at 37 °C. Subsequently, the cells were analyzed using the FACS system (BD Biosciences). Cell apoptosis was determined by FACS analysis (Annexin V-FITC/PI staining) according to manufacturer's instructions (Beyotime). A549 cells were first transfected with CENPF siRNA and collected 48 h later. After the wash step, cells were resuspended in annexin V buffer. Then 5 μL annexin V-FITC was added in the dark for 10 min and 5 μL propidium iodide for another 5 min in the dark. Subsequently, cells were subjected to flow cytometry (BD Biosciences) for assessment of apoptosis. In order to control the experimental data error, the experiments were performed at least three times.

### Colony formation assay

A549 cells were plated at a density of 1000 cells/well in triplicate and grown for 10 days after transfection 3 days. Subsequently, after fixed with 20 % methanol and stained with Giemsa (Sigma), the colonies were counted under a microscope.

### *In vivo* armpit xenograft lung cancer models

All of the surgical procedures were complied with the institutional guidelines. Female 4-week-old BALB/c-nude mice were purchased from Lingchang Biotech (Shanghai, China). A total of 20 nude mice were randomly assigned to control group (n=20) and experimental group (n=10). The armpit of right forelimb of mice were injected with A549 cells at a density of 2×10^7^ in 200 μL serum-free RPMI 1640. Finally, the mice were sacrificed after inoculation 38 days. Tumor volume was calculated as follows: volume = π/6 ​× length × width × width. The tumors were harvested and frozen at -80 °C at the end of the experiments for our further studies. All of the animal procedures were approved by the Ethics Committee of Qilu Hospital of Shandong University.

### Statistical analysis

All statistical data were analyzed using SPSS 22.0 (SPSS, Chicago, IL, USA), GraphPad 7.0 (GraphPad Software, San Diego, CA, USA). The quantitative data are shown as the mean ± standard deviation (SD). The significance of the difference between lung cancer and adjacent non-tumor groups using Student's t-test. A paired t test or Wilcoxon matched-pairs signed rank test was applied to compare the differences in CENPF expression between LUAD and matched normal groups. Mann-Whitney U test was used for comparison quantitative variables distributed non-normal between two groups. The overall survival (OS) and relapse free survival (RFS) rate were analyzed using Kaplan-Meier analysis with the log-rank test. Differences with *P* < 0.050 were considered statistically significant.

## Results

### CENPF is overexpressed and correlated with the prognosis of LUAD

**Figure 1:**
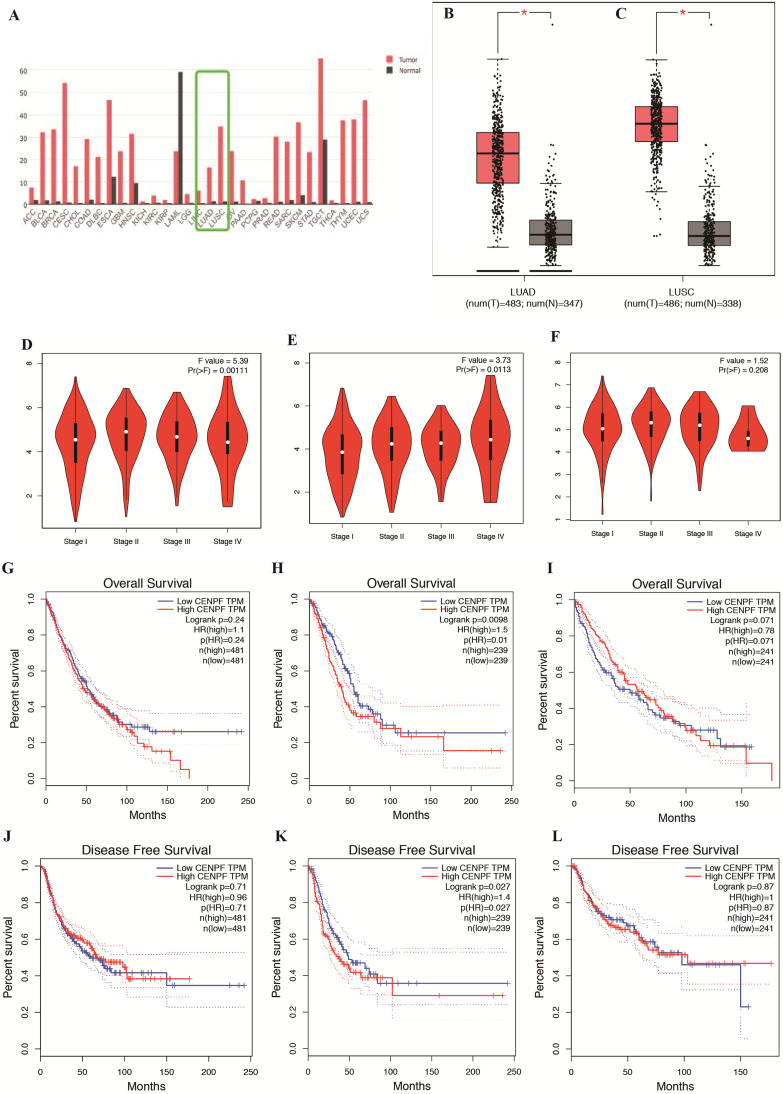
** CENPF is overexpressed in tumor tissues and its expression correlates with OS and RFS of LUAD patients. A-C,** Results from public database GEPIA indicated CENPF expression in LUAD and LUSC is higher than adjacent non-tumor lung tissues. **D-F,** CENPF mRNA expression was remarkably positively associated with pathological stage in NSCLC and LUAD, no significant difference of CENPF mRNA expression was found in LUSC patients. **G-I,** Online analysis of OS shows that higher CENPF expression indicates a poorer prognosis of LUAD, have no significance both in NSCLC and LUSC. **J-L,** Online analysis of RFS shows that higher CENPF expression indicates a poorer prognosis of LUAD, have no significance both in NSCLC and LUSC. * *P* < 0.05; ** *P* < 0.01; *** *P* < 0.001. CENPF, Centromere protein F; OS, overall survival; RFS, relapse free survival; LUAD, lung adenocarcinoma; GEPIA: Gene Expression Profiling Interactive Analysis; LUSC, lung squamous cell carcinoma; NSCLC, non-small cell lung cancer.

To explore the expression and potential role of CENPF in NSCLC (including LUSC and LUAD, separately), we first used the publicly available NSCLC database GEPIA to analyze CENPF mRNA expression between tumor specimens and normal tissues. As shown in Fig. 1A-C, CENPF mRNA expression was significantly elevated in both LUAD and LUSC tissues compared with adjacent non-tumor lung tissues (both *P* < 0.001). Furthermore, CENPF mRNA expression was remarkably positively associated with pathological stage in NSCLC (*P* = 0.001) and LUAD (*P* = 0.011), no significant difference of CENPF mRNA expression was found in LUSC patients (*P* = 0.208) (Fig. 1D-F). Most importantly, to explore whether CENPF mRNA expression levels will affect the clinical outcomes, we constructed a prognostic classifier using Kaplan-Meier analysis. As shown in the Fig. 1G-L, CENPF expression was significantly associated with both OS (*P* = 0.010) and RFS (*P* = 0.027) among the LUAD patients. As for NSCLC and LUSC, the expression levels of CENPF have no significance both with OS (*P* = 0.240 and *P* = 0.071) and RFS (*P* = 0.710 and *P* = 0.870) statistically.

**Figure 2:**
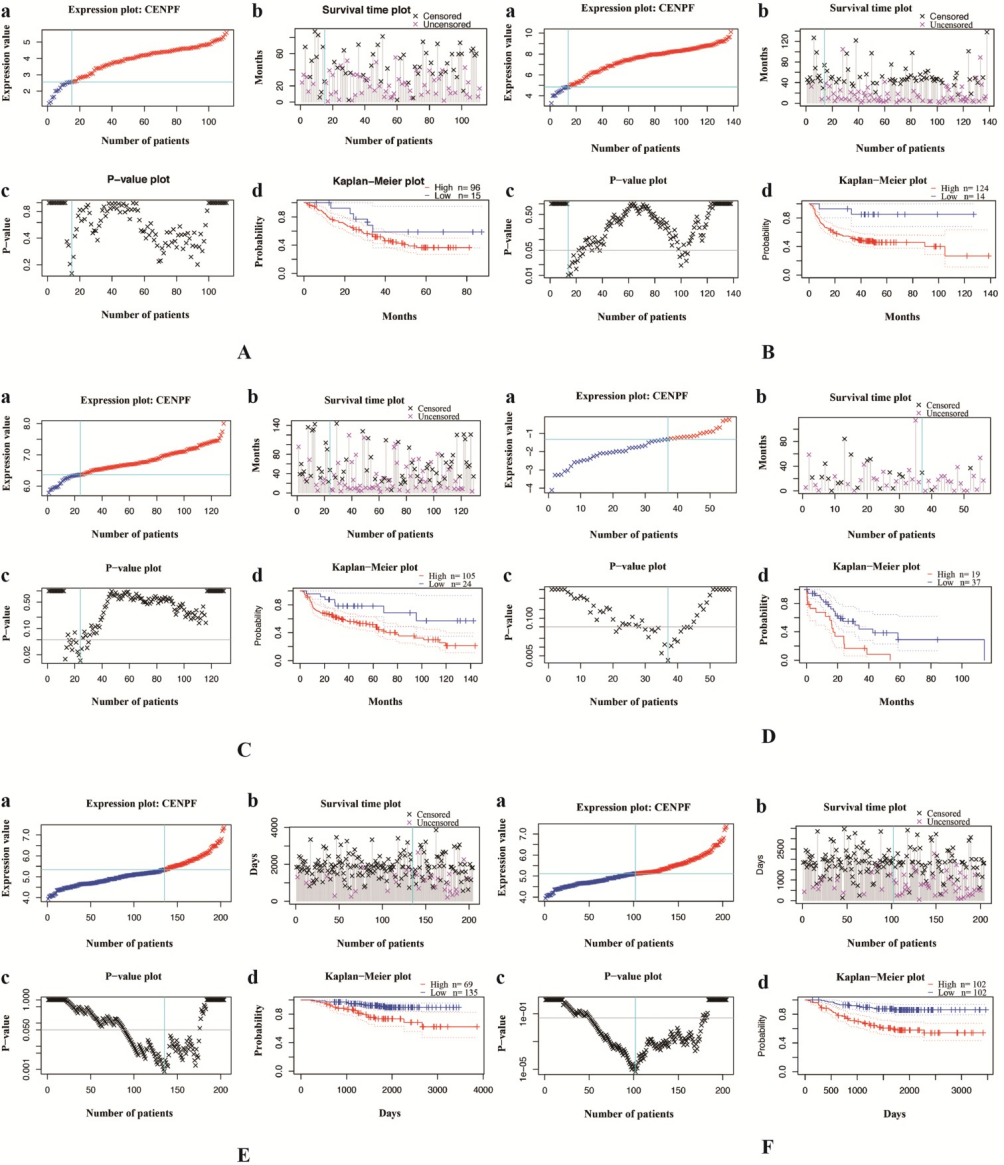
** Validation of the relationship between CENPF expression and the prognosis of NSCLC patients using independent cohorts from GEO database. A-B,** OS and RFS of NSCLC between low and high CENPF mRNA expression from GSE3141 and GSE8894. C-D, OS and RFS of LUSC between low and high CENPF mRNA expression from GSE4573 and GSE17710. E-F, OS and RFS of LUAD between low and high CENPF mRNA expression from GSE31210. **a,** Expression plot. Patients are ordered by the expression values of the given gene. The X-axis represents the accumulative number of patients and the Y-axis represents the expression value. Straight lines (cyan) show the optimal cut-points that dichotomize patients into high (red) and low (blue) expression groups. **b,** Survival time plot. Patients are ordered by the survival time of the given gene. The X-axis represents the accumulative number of patients and the Y-axis represents the survival time. **c,** P-value plot. For each potential cut-point of expression measurement, patients are dichotomized and survival difference between high and low expression groups is calculated by log-rank test. The X-axis represents the accumulative number of patients on the same scale as the expression plot and the Y-axis represents raw P-values on a log scale. The cut-point to minimize the P-value is determined and indicated by the cyan line. The gray line indicates the 5% significance level. **d,** Kaplan-Meier plot. Survival curves for high (red) and low (blue) expression groups dichotomized at the optimal cutpoint are plotted. The X-axis represents time and the Y-axis represents survival rate. 95% confidence intervals for each group are also indicated by dotted lines. CENPF, Centromere protein F; NSCLC, non-small cell lung cancer; GEO, Gene Expression Omnibus; OS, overall survival; RFS, relapse free survival; LUSC, lung squamous cell carcinoma; LUAD, lung adenocarcinoma.

**Figure 3:**
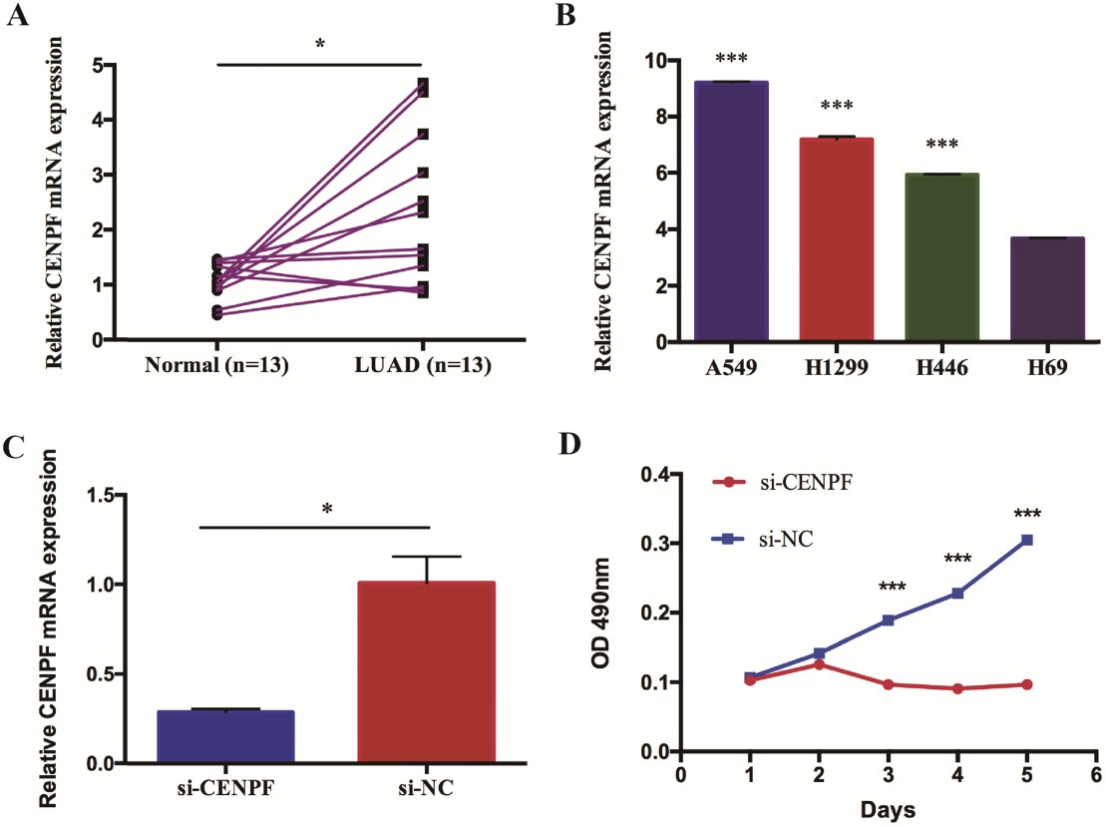
** CENPF promotes the proliferation of LUAD cell lines.** A, Quantitative real-time PCR (qRT-PCR) analysis of CENPF in 13 matched pairs of LUAD patients. B, CENPF expression was upregulated in the LUAD cells (A549 and H1299) compared with that of the SCLC cells (H69). C, qRT-PCR analysis of CENPF expression in A549 cells after transfection with a specifically synthesized siRNA. D, The MTT assay showed that CENPF promoted A549 cell proliferation. * *P* < 0.05; ** *P* < 0.01; *** *P* < 0.001. CENPF, Centromere protein F; LUAD, lung adenocarcinoma.

We validate the correlation between CENPF expression and the prognosis of NSCLC (including LUSC and LUAD, separately) patients using independent cohorts from GEO database. Due to the incomplete prognostic data of single gene expression profile, 5 different GEO datasets were retrieved for further validation. A total of 111 (GSE3141) and 138 (GSE8894) NSCLC patients, 129 (GSE4573) and 56 (GSE17710) LUSC patients, 204 (GSE31210) LUAD patients were used to validate the relationship between CENPF expression and OS as well as RFS, respectively. The results showed that CENPF mRNA expression have no significance with OS (*P* = 0.161 and *P* = 0.208) and RFS (*P* = 0.164 and *P* = 0.071) statistically both in NSCLC and LUSC (Fig. 2A-D). While the results showed that the hazard ratio (HR) of the mRNA expression of CENPF for OS was 1.720 with 95% confidence interval (CI) (1.060-2.770) (*P* = 0.022), for RFS was 1.770 with 95% CI (1.260-2.500) (*P* < 0.001) (Fig. 2E and F) in LUAD, suggesting that CENPF expression may be an indicator of the prognosis of LUAD patients. Besides, the expression of CENPF was verified in 13 matched pairs of LUAD tissues and adjacent non-tumor tissues using qRT-PCR. The results showed that CENPF was highly upregulated in LUAD (*P* = 0.010) (Fig. 3A), which was consistent with the microarray analysis results.

### CENPF promotes the proliferation of LUAD cell lines

To further explore the role of CENPF in LUAD, we performed a preliminary *in vitro* experiment. First, we examined CENPF expression in lung cancer cell lines. The results showed that CENPF was upregulated in the LUAD cell lines (A549 and H1299) compared with that in small cell lung cancer cell lines (H446 and H69) (Fig. 3B). Because CENPF was significantly upregulated in LUAD tissues and cell lines, it was considered as a target to investigate the role in LUAD tumorigenesis. A549 cells were transfected with si-CENPF or a negative control siRNA (si-NC). The qRT-PCR results revealed that CENPF expression was downregulated for 71.3% in LUAD cells by the siRNA compared with si-NC (Fig. 3C). To explore whether CENPF was involved in cell proliferation, we performed the MTT assay. We noticed that knockdown of CENPF greatly suppressed A549 cell proliferation (Fig. 3D). These *in vitro* experiments suggested that CENPF promoted LUAD cell proliferation.

### Impact of CENPF knockdown on LUAD cell cycle and apoptosis

**Figure 4:**
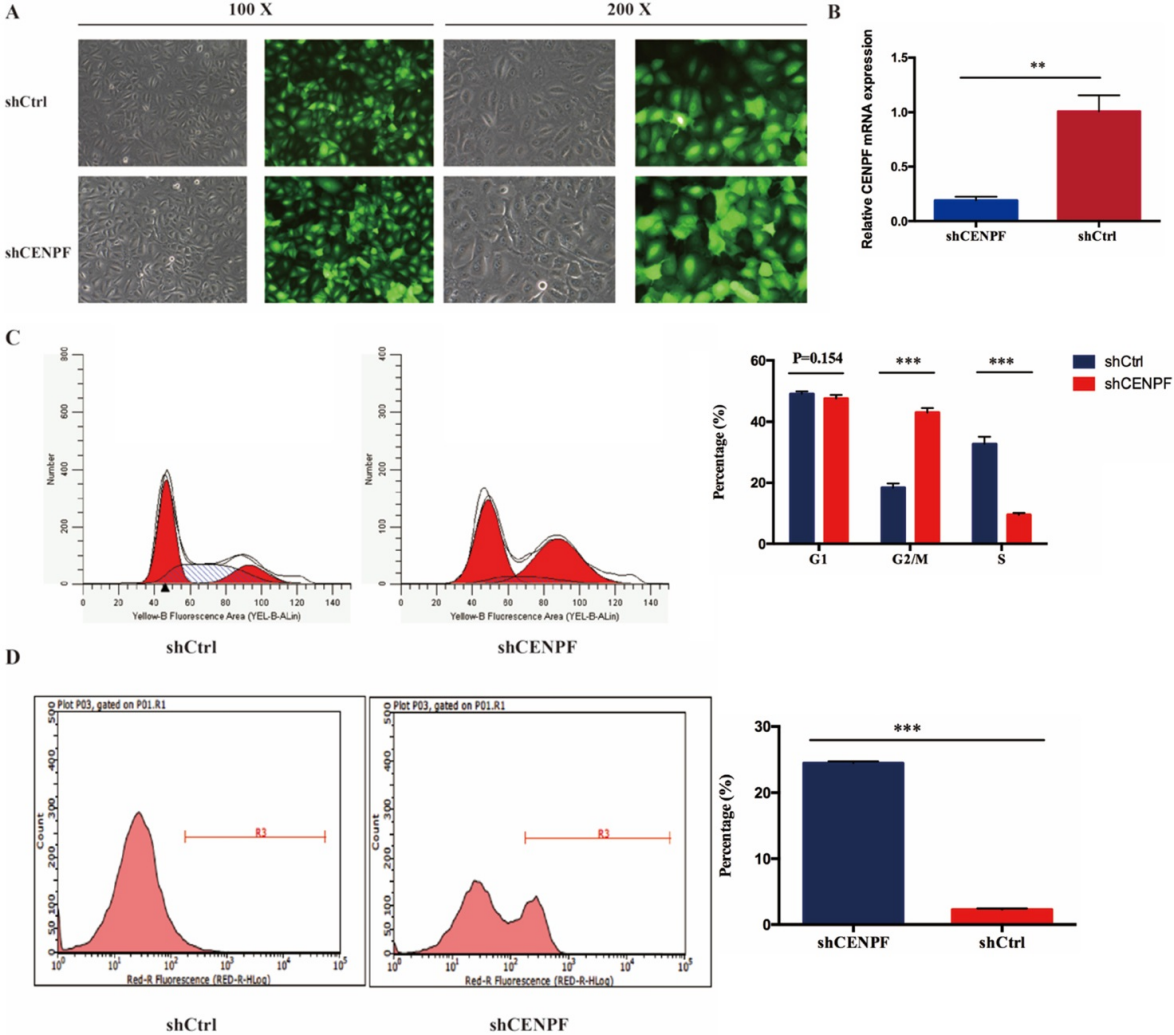
** Knockdown of CENPF in LUAD cells can block the cell cycle and induce apoptosis. A,** Visualization of the results were observed under fluorescence microscope after the lentivirus infection for 72 hours. **B,** Quantitative real-time PCR (qRT-PCR) analysis of CENPF expression in A549 cells after lentivirus infection. **C,** Knockdown of CENPF significantly impacted cell cycle distribution with induction of the G2/M and S phase fraction in A549 cell lines. **D,** The apoptosis rate in shCENPF cells was higher than that in the shCtrl groups. * *P* < 0.05; ** *P* < 0.01; *** *P* < 0.001. CENPF, Centromere protein F; LUAD, lung adenocarcinoma.

To evaluate the impact of CENPF knockdown on the proliferation of LUAD cell lines, A549 cells were infected with shCtrl and shCENPF lentivirus, respectively. After shCENPF lentivirus infection, the expression levels of CENPF mRNA were significantly decreased in the shCENPF group of A549 cells. The knockdown efficiency reached 80% (*P* < 0.010) (Fig. 4A). The qRT-PCR results revealed that CENPF expression was downregulated for nearly 80% in LUAD cells by the shCENPF lentivirus infection compared with shCtrl (*P* = 0.007) (Fig. 4B). CENPF knockdown significantly impacted cell cycle distribution with induction of the G2/M fraction (*P* < 0.001) as well as the S phase fraction (*P* < 0.001) the in A549 cell lines, while the percentage of cells in the G1 phase did not change significantly (*P* = 0.154). According to our results, we speculated that CENPF knockdown induced S phase arrest, which in turn contributes to the stimulating growth properties of LUAD cells (Fig. 4C). Cell apoptosis rate was quantified with FACS at 48 h after transfection. The apoptosis rate in shCENPF treated cells (24.48%) was higher than that in the shCtrl groups (2.29%) (*P* < 0.001) (Fig. 4D), which indicated that knockdown of CENPF promoted apoptosis in LUAD cells.

### Knockdown of CENPF inhibits cell growth of LUAD cells *in vitro* and *in vivo*

**Figure 5:**
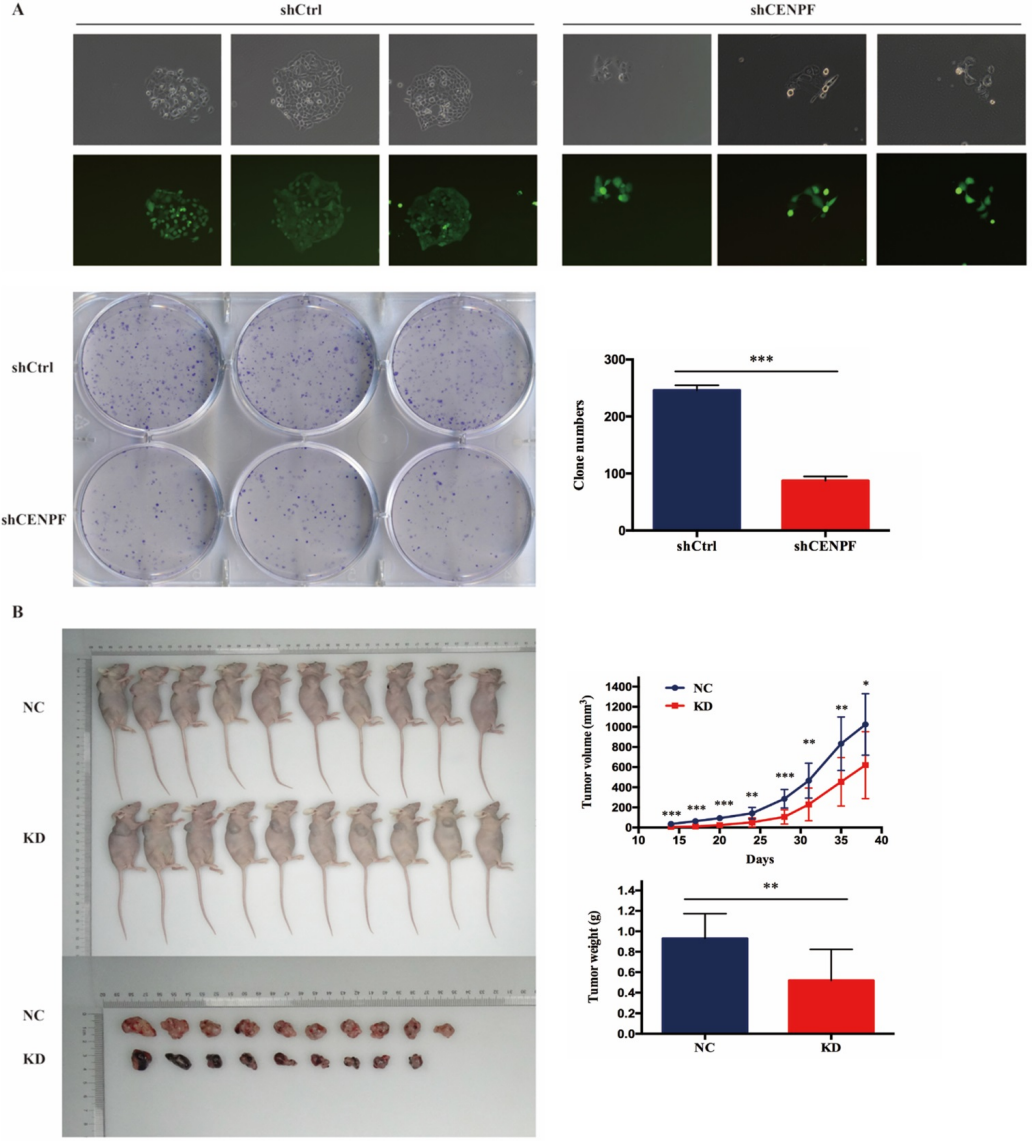
** Knockdown of CENPF expression inhibits cell growth of LUAD cells *in vitro* and *in vivo*. A,** The numbers of colony cells in the shCENPF groups significantly decreased compared with the shCtrl cells. **B,** The volume and weight of the tumors formed by the CENPF knockdown A549 cells and NC A549 cells. * *P* < 0.05; ** *P* < 0.01; *** *P* < 0.001. CENPF, Centromere protein F; LUAD, lung adenocarcinoma.

To confirm our data from CENPF knockdown, we also performed colony formation assay after A549 cells infected with shCtrl and shCENPF lentivirus. The results showed the numbers of colony cells in the shCENPF groups significantly decreased compared with the shCtrl cells (*P* < 0.001) (Fig. 5A). Cytofunctional *in vitro* experiments have proved that the knockdown of CENPF can inhibit the proliferation as well as promote the apoptosis of LUAD cells. To determine the effect of CENPF on LUAD cells tumorigenesis *in vivo*, we performed an experimental xenograft lung cancer model of nude mice armpit of right forelimb. At 14 days, 17 days, 20 days, 24 days, 28 days, 31 days, 35 days and 38 days after inoculation of A549 cells, the lung colonization of these cells in the A549-shCtrl and A549-shCENPF groups was monitored and quantitatively measured using a non-invasive bioluminescence system. The size (*P* < 0.050) and weight (*P* = 0.004) of the tumors formed by the A549-shCENPF cells significantly decreased in comparison with the tumors formed by the A549-shCtrl cells (Fig. 5B). Taken together, these results indicated that the knockdown of CENPF significantly inhibited the tumor growth of the LUAD cells *in vitro* and *in vivo*.

## Discussion

This study aimed to determine the role of CENPF in tumor growth and aggression in both clinical LUAD tissues and human LUAD cell lines. We compared the expression of CENPF in LUAD tissues and adjacent non-tumor tissues from the publically online database. Findings were also confirmed in 13 matched pairs of LUAD and adjacent tissues as well as lung cancer cell lines. Then we investigated the relationship between CENPF expression and clinical pathologic stage, at the meantime, we performed the OS and RFS rate of CENPF on LUAD patients using Kaplan-Meier analysis. *In vitro*, the A549 cell lines was selected for its highly expression and used to further explore cell biological behavior, including cell proliferation, colony formation, cell apoptosis as well as the changes of cell cycle. After CENPF knockdown, the cell proliferation of A549 cells slowed down, the cell cycle process was blocked, and the cell apoptosis rates increased, which indicated that CENPF could affect the function of A549 cells. Furthermore, Kaplan-Meier analysis results of OS and RFS showed CENPF could be used as a prognostic biomarker for LUAD.

CENPF is named after for its association with the centromere-kinetochore protein complex, while there are several studies reported its function in mitosis regulation and cellular proliferation 14, 15. As a novel therapeutic target, CENPF has been found to be overexpressed in various cancers and related with aggressive tumor phenotype as well as poor prognosis in different cancers 16. According to previous studies, MCM3AP-AS1 could hasten tumor growth in breast cancer by targeting CENPF via competitively binding to miR-28-5p 17. Besides, overexpression of hnRNPR promoted the aggressiveness of gastric cancer by increasing the mRNA expression of CCNB1 and CENPF 18. Maybe the molecular mechanisms underlying the effects of CENPF on the oncogenesis of LUAD also could be regulated by noncoding RNAs. However, the roles of CENPF in LUAD are less well understood and the functions of CENPF in LUAD has not been reported.

Cell proliferation and cell cycle are very important cellular processes, it could affect tumor formation and development, which controlled by numerous mechanisms, and the deregulation of cell cycle is a common feature of human cancers 19. CENPF was found related to the process of cell cycle and dynamically expressed throughout the cell cycle. Accumulating studies indicated high expression level of CENPF is associated with progress and prognosis in prostate cancer 20,21, breast cancer 22, hepatocellular carcinoma 23,24, esophageal squamous cell carcinoma 25 and nasopharyngeal carcinoma 26. CENPF knockdown can decrease HCC cells ability, form colonies and induce tumor formation in nude mice 27. Besides, as a downstream regulatory gene of MMP13, CENPF plays an important role in cell cycle, mitosis, and regulation of PLK1 activity in G2/M transition 28. Furthermore, different kinds of experiments have confirmed that the high expression of CEPNF is a prognostic indicator of survival and metastasis in ESCA patients 29,30. CENPF overexpressed was correlates with poor prognosis and tumor bone metastasis in breast cancer 31. In this study, we found that CENPF expression level in LUAD tissues was significantly elevated than adjacent non-tumor lung tissues using clinical samples. Besides, after CENPF knockdown in A549 cells, we performed vitro experiments to evaluate the role of CENPF in colony formation, cell cycle and apoptosis of lung cancer cell lines. The results showed that CENPF knockdown exerted a significant inhibitory effect on these processes, which means CENPF could affect the biological function of LUAD cells. Finally, we performed an experimental xenograft lung cancer model of nude mice armpit of right forelimb to determine the effect of CENPF on LUAD cells tumorigenesis *in vivo*. The size and weight of the tumors formed by the A549-shCENPF cells significantly decreased compared with A549-shCtrl cells. Taken together, our results indicated that CENPF was overexpressed both *in vitro* and *in vivo*, which was consistent with the results of previous studies on other cancers 32-36. Furthermore, high expression level of CENPF showed negative relationship with prognosis of LUAD in our study, which demonstrated the prognostic value of CENPF.

In conclusion, we demonstrated that CENPF was overexpressed in LUAD tissues and cell lines. *In vitro*, CENPF knockdown inhibited LUAD cell proliferation, cell cycle and apoptosis in LUAD cell lines. Besides, *in vivo* experiments showed the effect of CENPF tumorigenesis. All of our findings could be used as evidence supporting the role of CENPF, a potential prognostic biomarker for LUAD patients. However, there are still some limitations in our study, the deep insights into this phenomenon remain to be unclear, such as how to identify accurate molecules that could regulate the CENPF expression levels and the reason why high CENPF expression will correlated with poor prognosis of LUAD. These questions related to the potential molecular mechanisms were not explored experimentally. However, the results from our study indicated that CENPF could be a prognostic biomarker of LUAD.

## Abbreviations

LUAD: lung adenocarcinoma
CENPF: Centromere protein F
RFS: relapse free survival
OS: overall survival
qRT-PCR: quantitative reverse transcription-polymerase chain reaction
NSCLC: non-small cell lung cancer
LUSC: lung squamous cell carcinoma
GAPDH: Glyceraldehyde 3-phosphate dehydrogenase
STR: short tandem repeat
SD: standard deviation
HR: hazard ratio
CI: confidence interval
